# A genomic analysis of *Mycobacterium immunogenum* strain CD11_6 and its potential role in the activation of T cells against *Mycobacterium tuberculosis*

**DOI:** 10.1186/s12866-019-1421-y

**Published:** 2019-03-20

**Authors:** Gurpreet Kaur, Atul Munish Chander, Gurwinder Kaur, Sudeep Kumar Maurya, Sajid Nadeem, Rakesh Kochhar, Sanjay Kumar Bhadada, Javed N. Agrewala, Shanmugam Mayilraj

**Affiliations:** 1 Immunology Laboratory, CSIR- Institute of Microbial Technology, Chandigarh, India; 2 Department of Endocrinology, Post Graduate Institute of Medical Education and Research, Chandigarh, India; 3 Department of Biophysics, Panjab University, Chandigarh, India; 4 Microbial Type Culture Collection and Gene bank (MTCC), CSIR- Institute of Microbial Technology, Chandigarh, India; 5 Department of Gastroenterology, Post Graduate Institute of Medical Education and Research, Chandigarh, India; 6 Centre for Biomedical Engineering, Indian Institute of Technology, Ropar, India; 7 National Agri- Food Biotechnology Institute, Mohali, Punjab India; 8 Present address: Dr. Shanmugam Mayilraj, Bentoli Agri Nutrition Pvt. Ltd, Chennai, India

**Keywords:** *Mycobacterium immunogenum* (*Mi*), *Mycobacterium tuberculosis* (*Mtb*), *Mycobacterium bovis (Mb)*, Whole genome sequencing, Immune response, T cell memory

## Abstract

**Background:**

*Mycobacterium tuberculosis* (*Mtb*) is an etiological agent of tuberculosis (TB). Tuberculosis is a mounting problem worldwide. The only available vaccine BCG protects the childhood but not adulthood form of TB. Therefore, efforts are made continuously to improve the efficacy of BCG by supplementing it with other therapies. Consequently, we explored the possibility of employing *Mycobacterium immunogenum* (*Mi)* to improve BCG potential to protect against *Mtb*.

**Results:**

We report here the genome mining, comparative genomics, immunological and protection studies employing strain CD11_6 of *Mi*. *Mycobacterium immunogenum* was isolated from duodenal mucosa of a celiac disease patient. The strain was whole genome sequenced and annotated for identification of virulent genes and other traits that may make it suitable as a potential vaccine candidate. Virulence profile of *Mi* was mapped and compared with two other reference genomes i.e. virulent *Mtb* strain H37Rv and vaccine strain *Mycobacterium bovis* (*Mb*) AFF2122/97. This comparative analysis revealed that *Mi* is less virulent, as compared to *Mb* and *Mtb,* and contains comparable number of genes encoding for the antigenic proteins that predict it as a probable vaccine candidate. Interestingly, the animals vaccinated with *Mi* showed significant augmentation in the generation of memory T cells and reduction in the *Mtb* burden*.*

**Conclusion:**

The study signifies that *Mi* has a potential to protect against *Mtb* and therefore can be a future vaccine candidate against TB.

## Background

The treatment and control of tuberculosis (TB) is a growing challenge worldwide due to emerging multi-drug resistant strains of *Mycobacterium tuberculosis (Mtb),* failure of BCG vaccine and AIDS pandemic. BCG is a controversial vaccine since its protective efficacy varies from 0 to 85% [1–3]. Although, maximum number of people have been vaccinated with BCG worldwide, yet TB continues to afflict the global population. The vaccine protects the childhood but not adulthood manifestation of the disease. Extensive clinical trials conducted at Chengalpattu, Tamil Nadu, India suggested that BCG failed to protect TB-endemic population [4, 5]. Development of a safe and efficacious vaccine against TB has been recognized as an immediate global priority by the WHO. Alternative TB vaccines based on a BCG platform, or novel approaches to supplement BCG with agents that could enhance its efficacy, could be useful to combat TB in future. One of the many reasons for the failure of BCG vaccine is its inability to generate CD8 T cell response and to elicit long-lasting memory T cells [6, 7]. The earliest principles of vaccinology involves use of attenuated pathogens to allow its safe administration. Killed whole-cell mycobacterial vaccines may be a potential strategy for successful TB vaccine development. Seventy years ago, inactivated whole-cell mycobacterial vaccines administered in multiple-doses have shown protective efficacy in experimental models, as well as humans, but were not developed further after the discovery of BCG [8]. A prominent example is *Mycobacterium vaccae* (‘Vaccae’), a heat-inactivated whole-cell vaccine against TB, which is already being approved in China for the adjunct treatment of TB [9]. *Mycobacterium immunogenum (Mi)* is a non-tuberculous *mycobacterium* that is associated with hypersensitivity pneumonitis (HP). We did not observe any report demonstrating the role of *Mi* in protection against *Mtb*. The *Mi* strain CD11_6 was isolated from duodenal mucosa of a celiac disease (CD) patient. The strain was sequenced for determining its probable role in pathogenesis of CD, as microbes are known to play an important role in celiac autoimmunity [10–14]. The genomic annotations of *Mi* indicates that it possesses fewer virulence genes, as compared to BCG and target organism *Mtb.* Further, our *in-silico* studies indicate that it has sufficient antigenic repertoire; suggesting that it has enough potential to protect against *Mtb*. Sequence of *Mi* suggested that 16 out of total 26 peptides from *Mtb* showed sequence homology ranging 57–100% with similar cell surface proteins or secretory proteins of CD11_6 (*Mi)*. Further, prior published literature suggests a probable role of *Mi* in eliciting immune response [15, 16]. This preliminary information had derived our interest to explore protective efficacy of *Mi* against *Mtb*. Keeping in view of above mentioned facts, we adjudged to use heat killed *Mi* as a vaccine candidate and check its efficacy against *Mtb*. The present study indicates that *Mi* can induce optimum activation of both CD4 T cells and CD8 T cells. Further, enhancement in the pool of memory T cells was noticed, as indicated by modulation in the expression of the memory markers CD44, CD127 and CD62L. Consequently, this signifies a possible use of *Mi* as a vaccine candidate against *Mtb*.

## Results

### Characterization and phylogenetic analysis of strain CD11_6

**Fig. 1:**
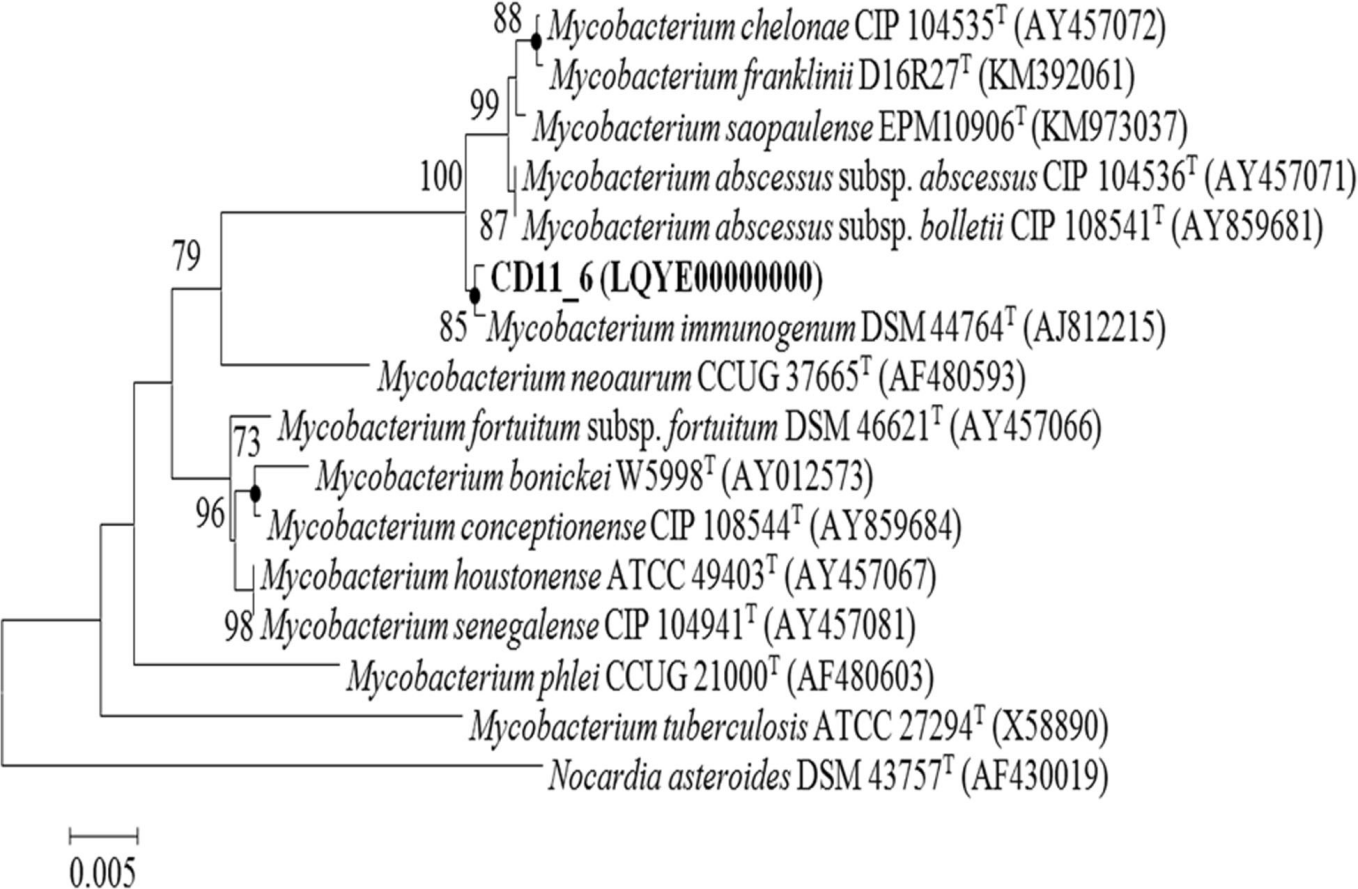
*Neighbour-joining tree based on 16S rRNA gene sequences, showing the phylogenetic relationship between the strain CD11_6 and other related members of the genus* *Mycobacterium*. *Nocardia asteroides* DSM 43757^T^ (AF430019) was used as an out group. Bootstrap values (expressed as percentage of 100 replications) greater than 70% are given at the nodes. Filled circles indicate that corresponding nodes were also recovered in the trees generated with maximum parsimony and maximum likelihood algorithms. Bar 0.01% sequence variation

The *Mi* strain designated as CD11_6 had matched with most of the phenotypic (Gram-positive, aerobic, rods in shape, acid fast, non-motile) characteristics of the genus *Mycobacterium* and most of the other features matched with the species *M. immunogenum (Mi):* negative for nitrate reduction, utilization of citrate; positive for D-glucitol, i-myo-inositol, and D-mannitol. Antimicrobial test of *Mi* showed susceptibility to amikacin, clarithromycin and resistant to cefoxitin, cefmetazole, ciprofloxacin, doxycycline, imipenem, sulfamethoxazole and tobramycin. 16S rRNA gene sequence showed that the strain CD11_6 correspond to the genus *Mycobacterium* and is most closely related to *M. immunogenum* CCUG 47286^T^ (99.93% identity) followed by *Mycobacterium abscessus* subsp. *bolletii* BD^T^ and *Mycobacterium abscessus* subsp. abscessus ATCC 19977^T^ (99.56%), *Mycobacterium saopaulense* EPM10906^T^ (99.49% identity), *Mycobacterium franklinii* CV002^T^ (99.42% identity), *Mycobacterium chelonae* ATCC 35752^T^ (99.42% identity) and *Mycobacterium salmoniphilum* ATCC 13758^T^ (99.42% identity). A collective phylogenetic tree comprising of Neighbor joining (NJ), Maximum likelihood (ML) and Maximum parsimony (MP) of strain CD11_6 was constructed using the 16S rRNA gene sequences of the most closely related species for the genus *Mycobacterium*. Strain CD11_6 formed a separate branch with *Mi* DSM 44764^T^ (Fig. 1).

### Genome features

The draft genome of strain CD11_6 consisted of 5,272,194 bp with G + C content 64.4 mol%, 5104 predicted CDS, 398 sub-systems and 59 RNAs. The final assembly contained 35 contigs with N_50_ contig length of 369,643 bp and the largest contig assembled was measured 1,437,452 bp. CD11_6 (*Mi*) genome was the largest (5.2 Mb) among the genome of *Mtb* (4.3 Mb) and *Mb* (4.4 Mb). Both strains *Mtb* and *Mb* had 65.6% genomic G + C content whereas *Mi* had 64.4%. Other genome features of strain CD11_6 along with two reference genomes are shown in Table 1.

**Table 1:** Genome features of strain CD11_6 and other reference strains of genus *Mycobacterium*

Organism	*Mycobacterium immunogenum* strain CD11_6	*Mycobacterium tuberculosis* strain H37Rv	*Mycobacterium bovis* strain AFF2122/97
Accession number	LQYE00000000.1	NC_000962.3	NC_002945.4
Isolation source	Duodenal mucosa of CD patient	Human sputum	Diseased Cow
Size (Mb)	5.27	4.41	4.35
Contigs	35	1	1
Scaffold	32	1	1
G + C	64.4	65.6	65.6
tRNA	56	46	45
Other RNA	3	22	2
Number of RNA’s	59	48	48
Genes	5112	4008	4001
Proteins	5000	3906	3918
Pseudogenes	50	30	33
Number of subsystem	398	400	401
Coding sequences	5104	4249	4175

### Genome comparison

#### Draft genome visualization using BRIG

By using BRIG software that works based on BLAST, a circular comparative map of whole genome was generated as concentric rings. This map shows genome sequence in the form of concentric rings. Darker areas of rings represent 100% sequence similarity with the reference genome but lighter (grey) areas represent 70% sequence similarity. Upper and lower thresholds for sequence identity were set as 90% and 70%, subsequently.

**Fig. 2:**
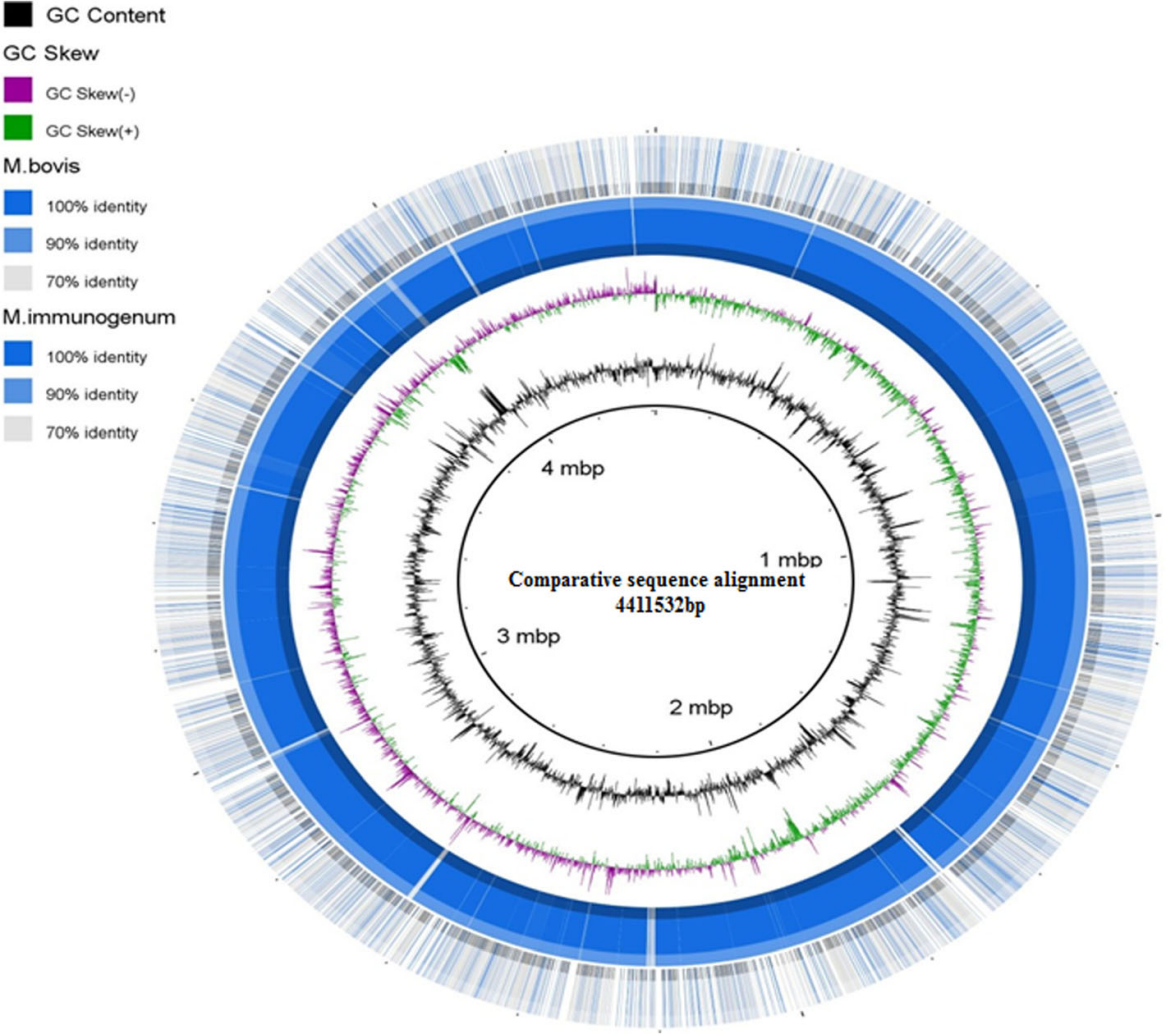
*Genome sequence comparison of all the three strains. *The innermost dark circle represents the reference genome of strain H37Rv (*Mtb),* the dark blue circle surrounding reference genome belongs to strain AFF2122/97 (*Mb)* and the outermost blue circle with white patches denotes genome of strain CD11_6 (*Mi*). The circles lying in between the reference genome and genome of strain AFF2122/97 (*Mb)* represents GC content (black coloured) and GC skew (dual coloured lines)

**Fig. 3:**
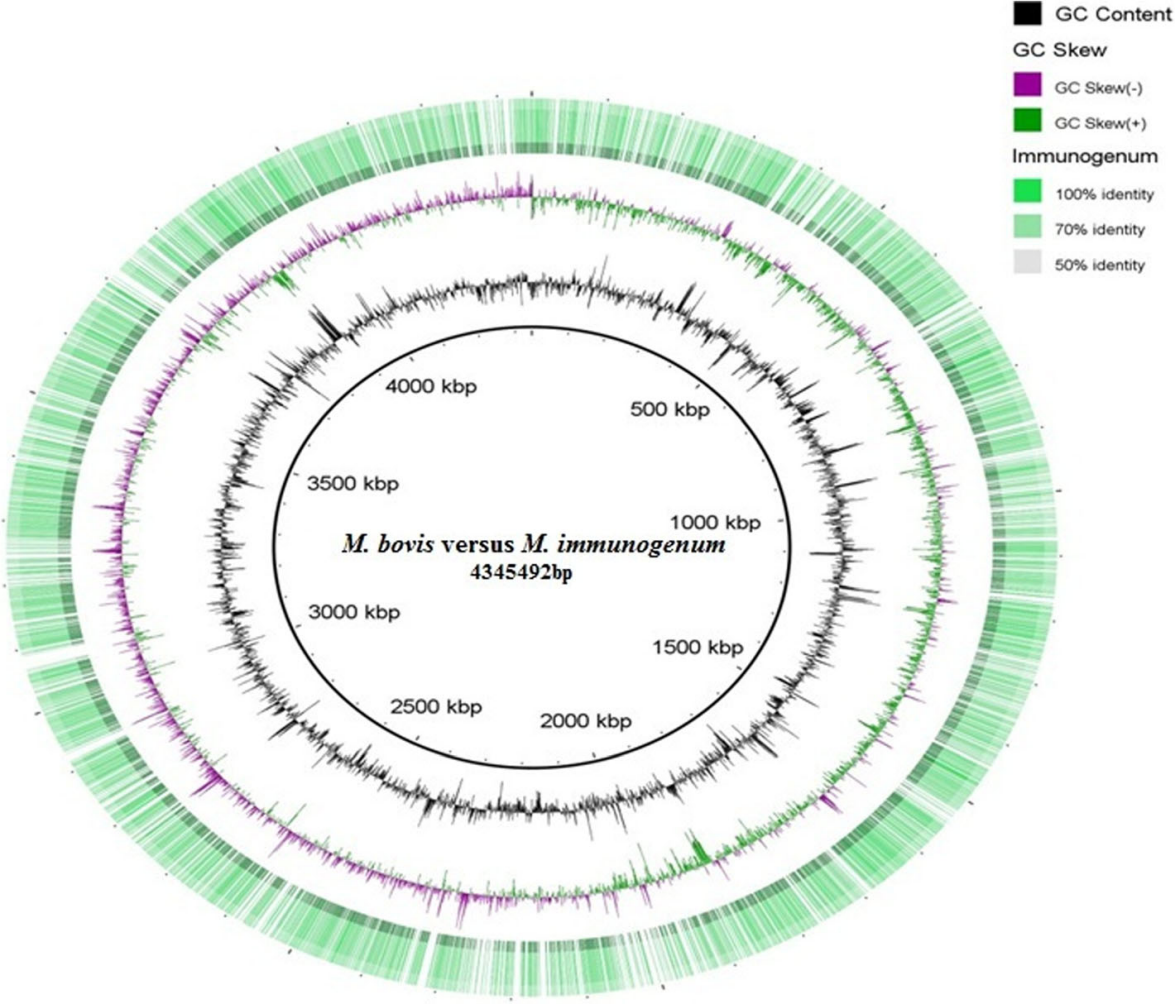
*Genome sequence comparison of* *Mb* and *Mtb*. Genome sequence comparison of strain AFF2122/97 (*Mb)* and strain CD11_6 (*Mi*). The innermost dark circle is the reference genome of strain AFF2122/97 (*Mb),* the outermost green circle denotes strain CD11_6 (*Mi*). Like Fig. 2, the circles lying in between the reference genome and genome of strain CD11_6 (*Mi*) represents GC content and GC skew

BRIG based sequence analysis among three strains was carried out. Initially, genome of H37Rv-*Mtb* was considered as the reference and two coloured concentric rings represent comparative level of similarity in genomes of strain AFF2122/97-*Mb* and strain CD11_6 *(Mi)* (Fig. 2). First dark blue concentric ring from centre represents genome of AFF2122/97-*Mb* and its sequence comparison with H37Rv-*Mtb,* whereas second concentric ring represents *Mi* and its sequence comparison with H37Rv-*Mtb.* Genome sequence of AFF2122/97-*Mb* resembles more closely with H37Rv-*Mtb,* as represented by the dark blue circle surrounding *Mtb* reference genome. However, genome sequence of *Mi* also shows sequence homology with genome of H37Rv-*Mtb, as* represented by several dark blue bands in the outermost circle. Therefore, it can be surmised that the genome of *Mi* may code for similar proteins like of *Mtb* and thus can be a potent vaccine candidate against *Mtb*. Similarly, Fig. 3 represents the comparative sequence analysis of strain CD11_6 *(Mi), with* strain AFF2122/97-*Mb* as a reference. Light bands in the concentric ring represent levels of dissimilarity whereas the dark coloured patches represent similarity with the reference genome. The map shows that genome sequence of *Mi* is very similar to that of AFF2122/97-*Mb,* already a vaccine strain being used against *Mtb.* BRIG map indicated genomic similarities but actual predictions can be attained by knowing about the coded proteins. Thus, the genomic similarities were further validated by annotating genomes of all the strains in RAST server and a comparison was established.

#### Comparison of relevant virulence determinants

In RAST server, genes are grouped into categories according to their major role in living systems. As per specific functions of genes, each category is divided into sub-categories which are further divisible into subsystems with a set of genes. Genome comparison of strain CD11_6 *(Mi),* was carried out with strains H37Rv-*Mtb* and AFF2122/97-*Mb*. The analysis revealed various categories of genes, among which virulence, disease and defence (VDD), iron acquisition and metabolism, membrane transport proteins, genes of phages, prophages and transposable elements, genes involved in stress response were further studied because of their extreme importance to evaluate the status of strain CD11_6 as a vaccine candidate. As per relevance, we studied two subcategories of VDD i.e. i] resistance to antibiotics and toxic compounds; ii] invasion and intracellular resistance.

#### Genes involved in virulence disease and defence (VDD)

For the category VDD, a total of 66 genes were present in the three genomes. Thirty six genes were common in three genomes. Interestingly, H37Rv-*Mtb* and AFF2122/97-*Mb* had 61 genes but CD11_6 had 41 genes. Thus, CD11_6 lacks 21 virulence factor genes of category VDD, compared to other two strains but contains almost common membrane proteins with H37Rv and AFF2122/97 (Table 2). The fact is in favour of *Mi* as an attenuated vaccine candidate that has lesser virulence factors than the target pathogen but has comparable number of same membrane proteins like the target organism (*Mtb*) and the well-established vaccine strain (*Mb*). In addition to the common membrane proteins, the strain CD11_6 *(Mi)* contains 3 unique membrane proteins whereas strain AFF2122/97 (*Mb)* had 2 unique genes. Subcategory “invasion and intracellular resistance” of VDD is most relevant because of the membrane associated proteins responsible for virulence that can act as vaccine candidate proteins. In all 47 genes in this subcategory, H37Rv had 10 genes coding for membrane proteins (Table 2). Strain AFF2122/97 had total 9 of these genes. Strain CD11_6 *(Mi)* had 6 genes common with AFF2122/97 and H37Rv. Thus, CD11_6 *(Mi)* contains almost similar membrane proteins like the target organism and *Mb*. In the next sections, we predicted the potential of strain CD11_6 *(Mi),* as a suitable candidate for vaccination against *Mtb*.

**Table 2:** Representation of common membrane proteins in all organisms

S.No	Name of membrane proteins	*Mi*	*Mtb*	*Mb*
1.	Inner membrane protein translocase component YidC, long form	Y	Y	Y
2.	Conserved hypothetical integral membrane protein YrbE1A	Y	Y	Y
3.	Conserved hypothetical integral membrane protein YrbE1B	Y	Y	Y
4.	MCE-family lipoprotein LprK (MCE-family lipoprotein Mce1e)	Y	Y	Y
5.	MCE-family protein Mce1B	Y	Y	Y
6.	Fig. 025093: Probable membrane protein	Y	Y	Y
7.	Fig. 030769: Probable conserved MCE associated membrane protein	N	Y	Y
8.	Type VII secretion integral membrane protein EccD	N	Y	N
9.	Fig. 033285: Conserved MCE associated transmembrane protein	N	Y	Y
10.	Fig. 033430: Probable conserved MCE associated membrane protein	N	Y	Y

Y: presence of a gene; N: absence of a gene

#### Genes involved in invasion and intracellular resistance

**Fig. 4:**
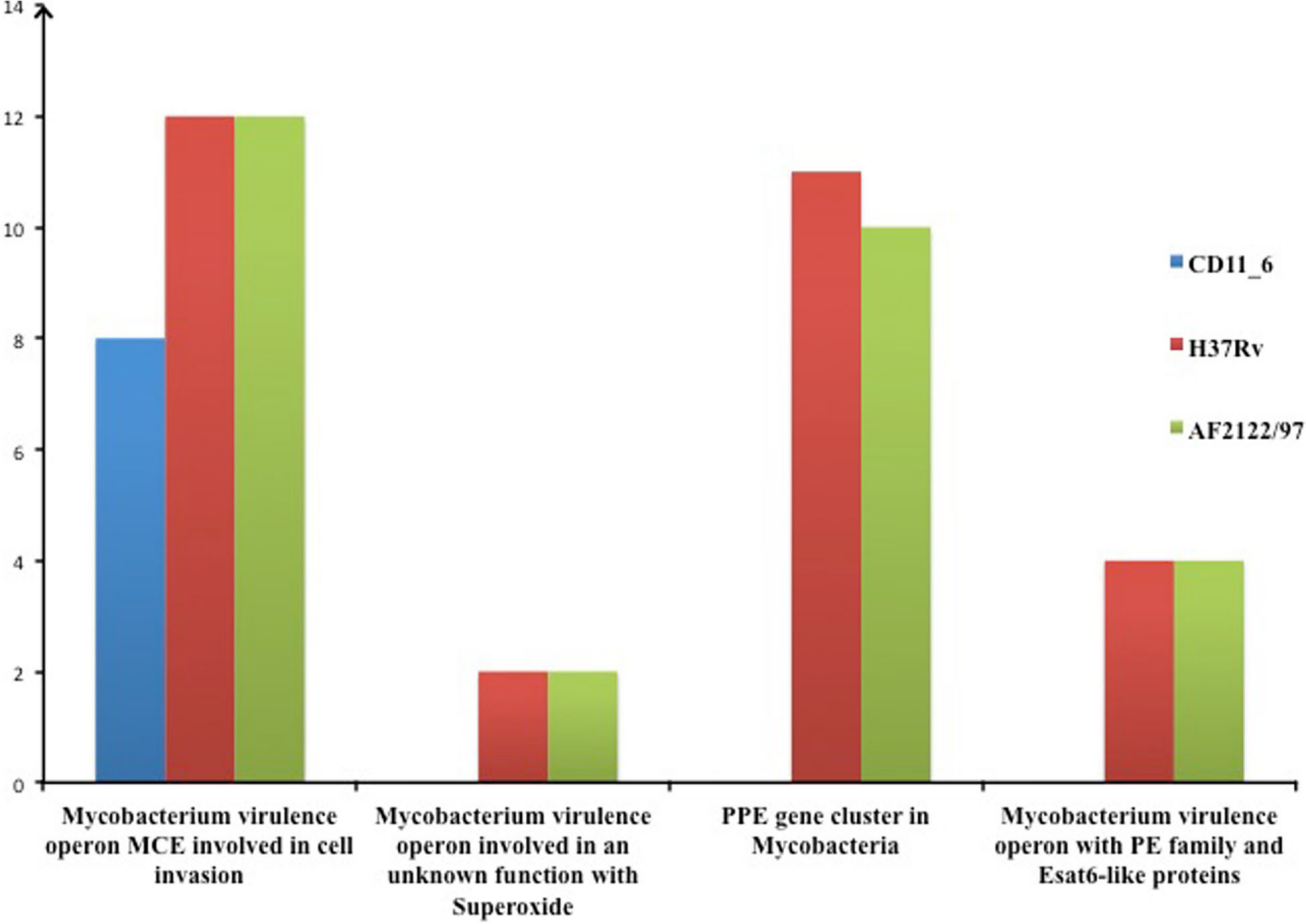
*Comparison of gene homologues present in the Mycobacterium strains involved in invasion and intracellular resistance.* Genetic variability was reported in subsystems *Mycobacterium* virulence operon MCE involved in cell invasion, *Mycobacterium* virulence operon involved in an unknown function with Superoxide dismutase and PPE gene cluster in *Mycobacteria* whereas for other subsystems no such difference was reported among three genomes. Thus, for all three genomes, 2 genes were present in subsystem *Mycobacterium* virulence operon involved in an unknown function with a Jag Protein and YidC and YidD, subsystem *Mycobacterium* virulence operon possibly involved in quinolinate biosynthesis had 3 genes, *Mycobacterium* virulence operon involved in DNA transcription had 2 genes, subsystem *Mycobacterium* virulence operon involved in protein synthesis (SSU ribosomal proteins) had 5 genes, subsystem *Mycobacterium* virulence operon involved in fatty acids biosynthesis had 2 genes, subsystem *Mycobacterium* virulence operon involved in lipid metabolism had 2 genes, subsystem *Mycobacterium* virulence operon involved in protein synthesis (LSU ribosomal proteins) had 3 genes

In this sub-category, strain H37Rv-*Mtb* had 48 genes, strain AFF2122/97-*Mb* had 46 genes and strain CD11_6 *(Mi)* had 27 genes. Out of total 48 genes, 27 are common in all. Unlike other two strains, CD11_6 *(Mi)* does not contain any gene for PPE gene cluster, *mycobacterium* virulence operon with PE family; ESAT-6-like proteins, and *mycobacterium* virulence operon in an unknown function with superoxide dismutase (Fig. 4). For all other gene functions, strain CD11_6 *(Mi)* had equal or less genes compared to strain H37Rv-*Mtb* and AFF2122/97-*Mb* supporting the low virulence attributes of strain CD11_6.

PPE proteins, early-secreted antigen target (ESAT-6) like proteins and the 10-kDa culture filtrate protein (CFP10) are the product of region of deletion 1 (RD1) [17]. RD1 codes for 9 genes and most of them are responsible for virulence and pathogenesis [18].

In the sub-category, invasion and intracellular resistance, the presence of subsystem *mycobacterium* virulence operon was observed in *Mtb* and *Mb* that contains ESAT-6- like proteins (ESAT-6-like protein EsxL and ESAT-6-like protein EsxK), whereas *Mi* lacks such genes. During BLAST search analysis in the genomes, 6 kDa early secretory antigenic target ESAT-6 (EsxA) was also reported to be present in *Mtb* and *Mb* but not in *Mi.* These associated genes are responsible for virulence in *Mtb* [19]. The presence of CFP-10 was observed both in *Mtb* and *Mb* but not in *Mi*. The lack of such genes in *Mi* validates it even safer than *Mb.*

#### Genes involved in resistance to antibiotics and toxic compounds

**Fig. 5:**
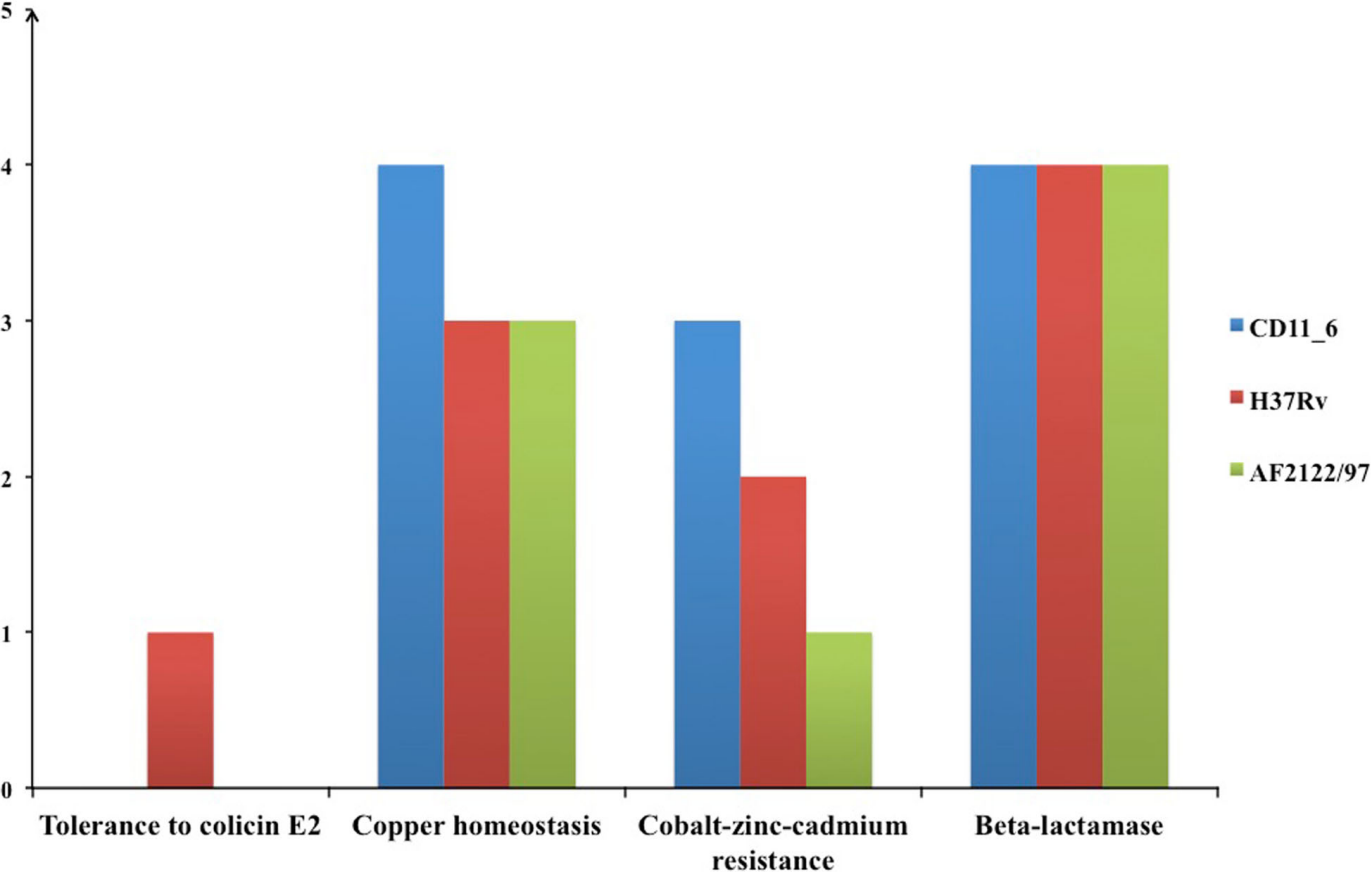
*Genes involved in subcategory resistance to antibiotic and toxic compounds.* The subsystem Mercuric reductase had one gene present in all the organisms and subsystem resistance to fluoroquinolones had two genes in all three microbes, whereas the subsystems showing difference are represented in this figure

Genes responsible for resistance to antibiotics and toxic compounds enable organisms to survive in adverse clinical environments. Strain CD11_6 *(Mi)* encoded 14 genes, H37Rv-*Mtb* had 13 genes and AFF2122/97-*Mb* had 11 genes in this subcategory (Fig. 5). Like other strains, CD11_6 possess same 2 genes that can cause resistance to fluoroquinolones. CD11_6 *(Mi)* had 4 genes that can be responsible for resistance against beta-lactam antibiotics and 3 of these genes were also common in other 2 strains*.* Gene coding for metal-dependent hydrolases of the beta-lactamase superfamily III is unique in CD11_6*,* whereas gene coding for enzyme beta-lactamase class A was absent in CD11_6*,* but common in strains AFF2122/97-*Mb* and H37Rv-*Mtb.*

#### Predictions for immunization potential of *Mi* CD11_6 against *Mtb*

Immunization potential of strain CD11_6 *(Mi)* against H37Rv-*Mtb* was predicted using computational approaches. Twenty six vaccine candidate peptides as described by Carolina Vizcaino *et.al.* were considered for our *in-silico* comparisons [20]. These peptides belonged to membrane proteins or secretory proteins in different strains of *Mtb*. Sixteen of these 26 peptides showed sequence homology ranging 57–100% with similar cell surface proteins or secretory proteins of CD11_6 *(Mi)* (Table 3). Our previous data showed that CD11_6 *(Mi)* and H37Rv have similar membrane proteins. In this section, similarity percentages indicate that the proteins encoded by strain CD11_6 *(Mi)* also contains similar vaccine peptides like those derived from different strains of H37Rv-*Mtb* by Carolina Vizcaino *et.al.* These sequence homology data may support the potential of strain CD11_6 *(Mi)* as a vaccine candidate against *Mtb*.

**Table 3:** Sequence homology between vaccine candidate peptides of *Mtb* and *Mi*

S.No	Strains of *Mycobacterium tuberculosis*	Vaccine candidate peptides of secretory and membrane proteins	Similar peptides as vaccine candidates in *Mi*	Name of the protein these peptides belong	Sequence homology
1.	Rv0200 (+)	LVLLVVEGVAINFWLLRRD	^45^LLITQGMAINAYLARRD^61^	Possible membrane protein, Rv0200	14/17 (82%)
2.	Rv0200 (+)	QAARALRVTLTKRGSGWLV	^203^QSVLALRLTLAKHDGRWLV^221^	Possible membrane protein, Rv0200	12/19 (63%)
3.	Rv1280C (+)	DGYQDNSGVVAYNPEQAKRE	^780^DGYQDEWAAVLADPDKLRR^798^	Nitrite reductase [NAD(P)H] large subunit	12/19 (63%)
4.	Rv3630	LTRAPLLVPLTAMQGNLIAH	^271^ LTRAPLLVPLTALQGNLIAH^290^	Possible membrane protein	20/20 (100%)
5.	Rv3630	LTRAPLLVPLTAMQGNLIAH	^4^LTPLPVLIPLTAAALTLIA^22^	Na(+) H(+) antiporter subunit D	13/19 (68%)
6.	Rv0418 (+)	LKMAGKTAQDTSFDGRSDYD	^388^LAEQGKKAHDTGYDGRSDYD^407^	Aminopeptidase Y (Arg, Lys, Leu preference) (EC 3.4.11.15)	14/20 (70%)
7.	Rv0418 (+)	LKMAGKTAQDTSFDGRSDYD	^389^LNLAGKRPAAEEFSGRSDY^407^	Aminopeptidase Y (Arg, Lys, Leu preference) (EC 3.4.11.15)	11/19 (57%)
8.	Rv0418 (+)	VAAPADDSPGCSPSDYDRLP	^144^VPARAEESPGCTVEDYDGL^162^	Aminopeptidase Y (Arg, Lys, Leu preference) (EC 3.4.11.15)	14/19 (73%)
9.	Rv0418 (+)	VAAPADDSPGCSPSDYDRLP	^28^AAADDPPNCTPAD^40^	Exported protein	10/13 (76%)
10.	Rv1022	DGIANVDNIDDAALSAAGYL	^193^DGEPSPDNIDDAALSAAGYL^212^	Lipoprotein LpqU	17/20 (85%)
11.	Rv0566 (+)	TPDRITYRPQLGVLYPSELS	^51^TPERVTYRPELGVLYPSELS^70^	Possible membrane protein	20/20 (100%)
12.	Rv0566 (+)	TPDRITYRPQLGVLYPSELS	^177^DAIAYLPEFGVLYAGDL^193^	Fumarylacetoacetase (EC 3.7.1.2)	11/17 (64%)
13.	Rv0361	DAETETVVITTSDNDAAVTQ	^189^SEGRTVVLTTHDMDEAVS^206^	ABC transporter, ATP-binding protein	13/18 (72%)
14.	Rv0361	RSLDLQFRDDQWKITQSSSN	^242^RSFDLQFRDNQWKICQS^258^	Probable conserved membrane protein	15/17 (88%)
15.	Rv1326c (−)	MSRSEKLTGEHLAPEPAEMA	^348^ERLTVEHALPSPAEL^362^	NTD biosynthesis operon putative oxidoreductase NtdC	11/15 (73%)
16.	Rv1326c (−)	RFDGTPLYEHSDPKRGEQLD	^359^RFDGTPLYEHADPHRAEQLD^378^	1,4-alpha-glucan (glycogen) branching enzyme, GH-13-type (EC 2.4.1.18)	18/20 (90%)

#### Immunization with *Mi* increases the pool of activated T cells and reduces the *Mtb* burden in the lungs and spleen

**Fig. 6:**
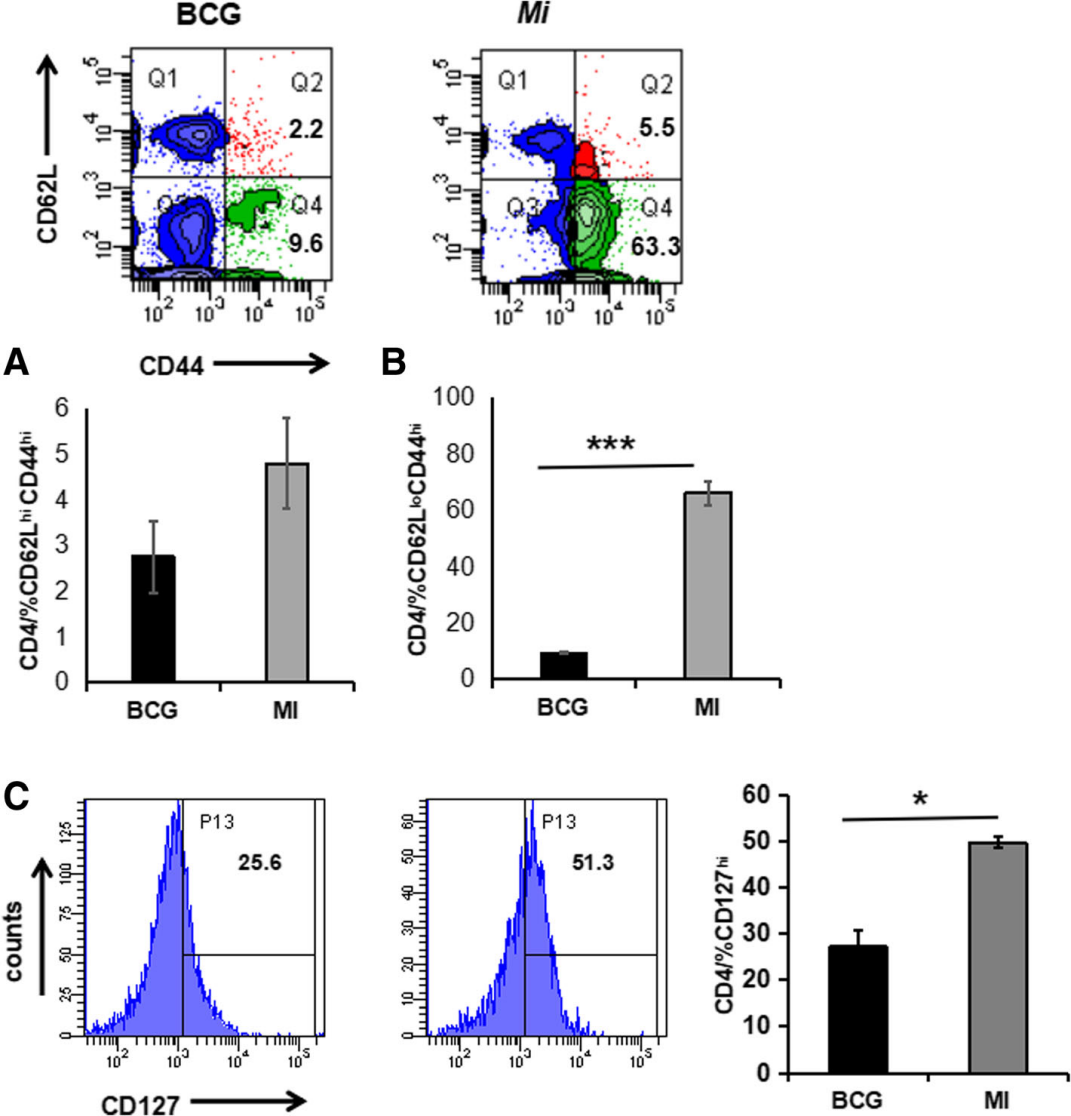
*Immunization with Mi augments the pool of CD44hi, CD62Llo and CD127hi expressing memory CD4 T cells.* Lymphocytes isolated from the lungs of mice immunized with *Mi* and infected with *Mtb* were cultured in vitro with PPD. The control group of mice were inoculated with BCG. The cells were then monitored for the expression of activation markers (**a**) CD62L^hi^CD44^hi^; (**b**) CD62L^lo^CD44^hi^; (**c**) CD127^hi^ on CD4 T cells by flow cytometry. Increase in the percentage of CD62L^hi^CD44^hi^, CD62L^lo^CD44^hi^ and CD127^hi^ is depicted as flow cytometry histograms (left panel) and bar diagram (right panel). The data are representative of pooled lymphocytes of 4–5 mice/group obtained from triplicate wells. *p < 0.05, ***p < 0.0005

Both memory CD4 T cells and CD8 T cells play a fundamental role in protection against intracellular pathogens like *Mtb* [21, 22]. CD44^hi^, CD62L^hi^ and CD127^hi^ are known to be key molecules displayed on the surface of memory T cells [23]. As compared to BCG, we observed that the cells obtained from the animals exposed to *Mi* exhibited significant enhancement in the percentage of CD62L^hi^CD44^hi^, CD62L^lo^CD44^hi^ (*p* < 0.0005), CD127^hi^ (*p* < 0.05) and expressing CD4 T cells (Fig. 6), thus indicating that *Mi* induces the generation of central and effector memory CD4 T cells. CD62L^hi^CD44^hi^ phenotype represents central memory, whereas CD62L^lo^CD44^hi^ population represents effector memory [24]. CD4 T cells play a cardinal role in maintaining enduring protection against infection. BCG is known to induce diminished frequency of memory T cells which may be one of the reasons for its failure to impart long-lasting protection against *Mtb* [6]. These results suggest an important role of *Mi* in elicitation of memory CD4 T cells.

**Fig. 7:**
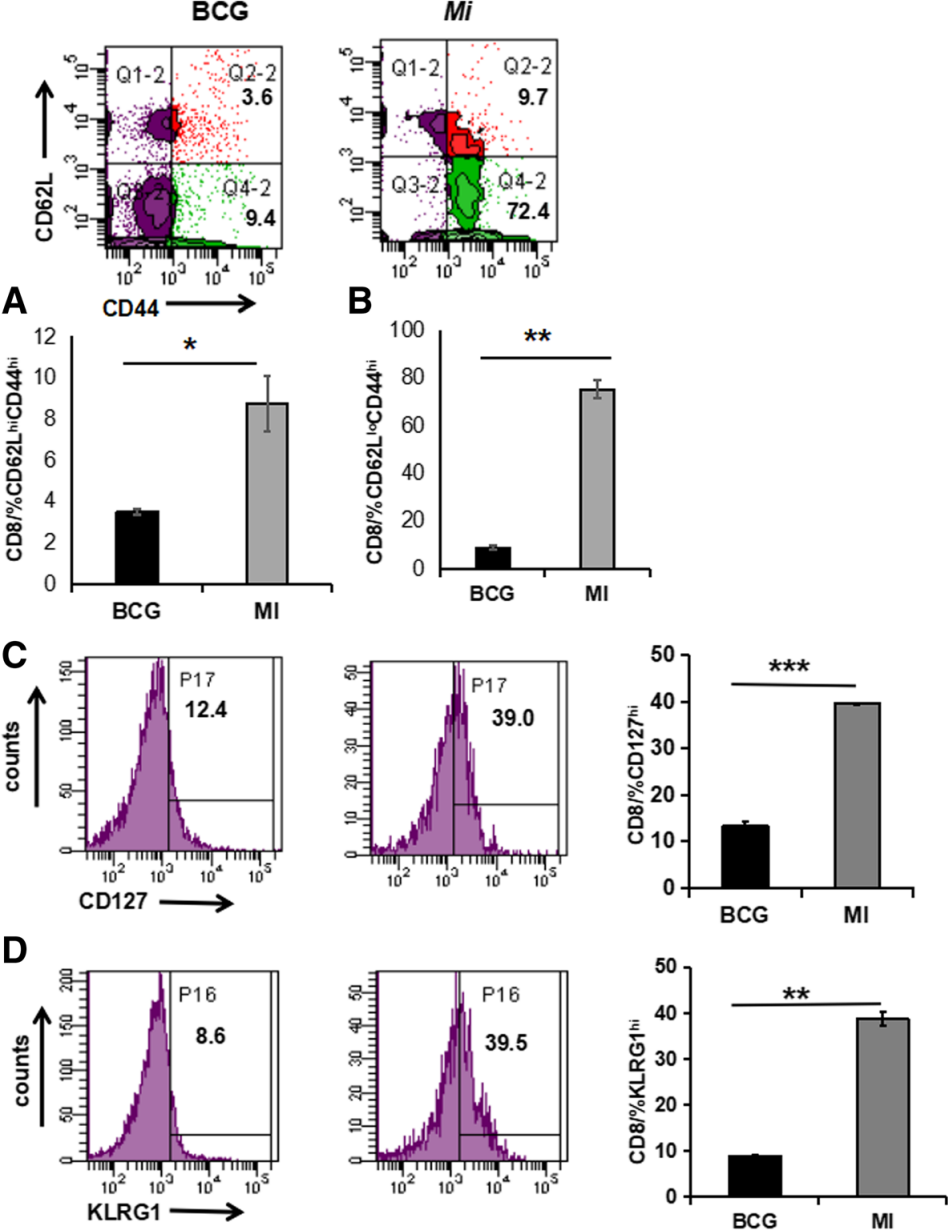
*Inoculation of Mi in animals expands the frequency of effector and central memory CD8 T cells*. Lymphocytes isolated from the lungs of mice injected with *Mi* and exposed to *Mtb* were cultured in vitro with PPD. The control group of mice was inoculated with BCG. The cells were then monitored for the expression of activation markers (**a**) CD62L^hi^CD44^hi^; (**b**) CD62L^lo^CD44^hi^; (**c**) CD127^hi^ (**d**) KLRG1^hi^ on CD8 T cells by flow cytometry. Increase in the percentage of CD62L^hi^CD44^hi^, CD62L^lo^CD44^hi^, CD127^hi^ and KLRG1^hi^ is depicted as flow cytometry histograms (left panel) and bar diagram (right panel). The data are representative of pooled lymphocytes of 4–5 mice/group obtained from triplicate wells. *p < 0.05, **p < 0.005, ***p < 0.0005

It has been demonstrated that β2-microglobulin knock-out mice, which are deficient in MHC class I molecules die rapidly of *Mtb* infection, thus signifying a crucial role of CD8 T cells in protection against *Mtb* [25–27]. Therefore, we next monitored the expansion in the pool of memory CD8 T cells in the animals immunized with *Mi*. Interestingly, in the case of CD8 T cells, both effector (CD62L^lo^CD44^hi^) and central memory (CD62L^hi^CD44^hi^) responses were significantly increased in *Mi* immunized animals as compared to animals immunized with BCG (Fig. 7). Further, a significant increase (*p* < 0.0005) in the percentage of CD127^hi^ expressing CD8 T cells was also observed in *Mi* immunized animals. Evidence supports the fact that expression of CD127 on CD8 T cells indicates a subset of effector CD8 T cells that successfully develop into fully protective memory. The combination of surface staining for CD127 and CD62L further separates between two functionally distinct memory cell subsets, central memory T cells (CD127^hi^ and CD62L^hi^) and peripheral effector memory T cells (CD127^hi^ and CD62L^lo^) [28, 29]. The phenotype observed in the *Mi* immunized CD8 T cells is an indicative of central memory, the cells responsible for long-term protection.

**Fig. 8:**
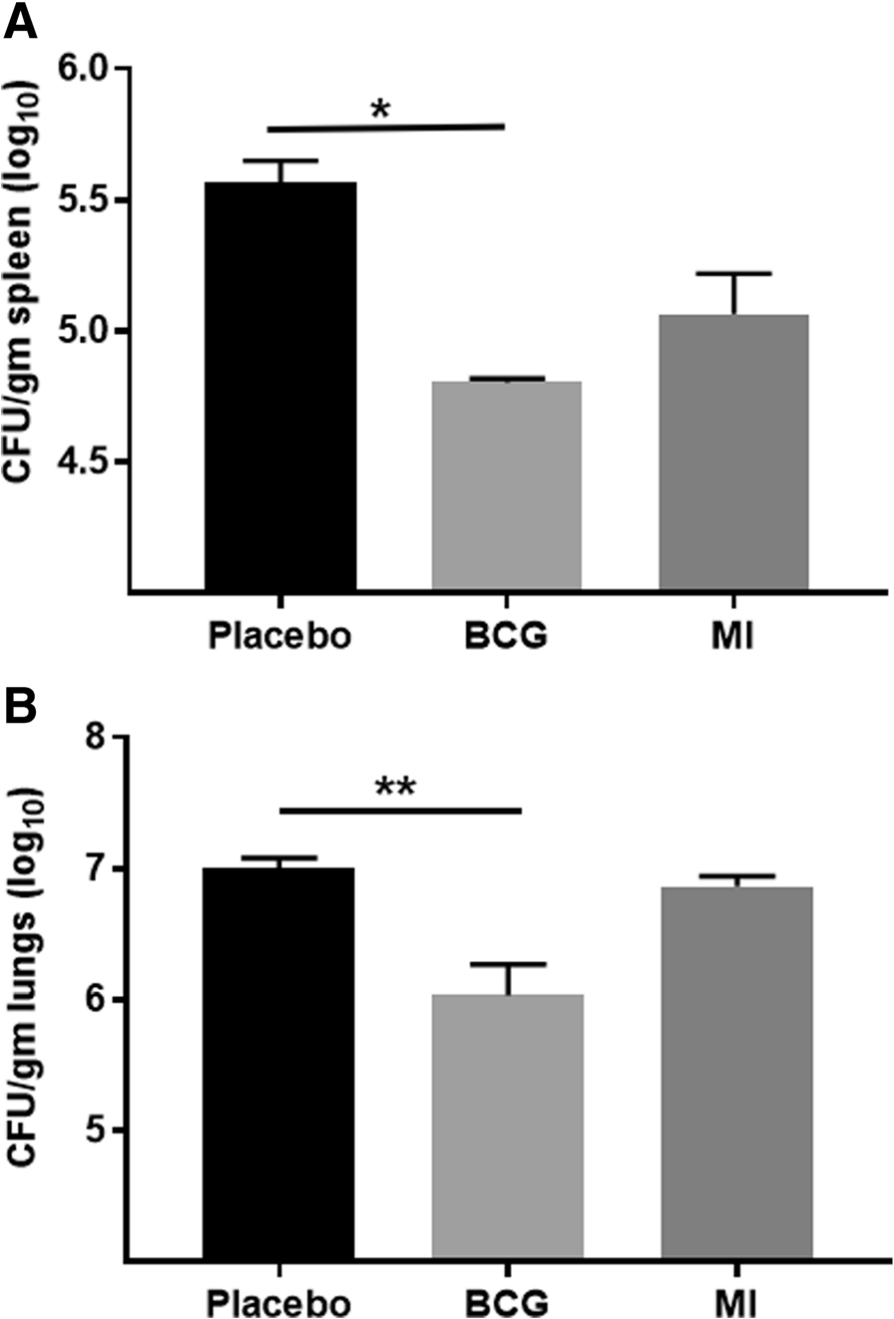
*Immunization with Mi reduces the Mtb burden in the lungs and spleen.* Mice (4 animals/group) were immunized with *Mi* (1 × 10^6^ bacilli/animal). The control groups were inoculated with BCG (positive control) and placebo PBS (negative control). After 30 days, animals were aerosol challenged with live *Mtb*. After 45 days of infection, animals were sacrificed and protective efficacy of immunization was determined by calculating bacterial burden in (**a**) spleen; (**b**) lungs by colony forming units (CFUs). Data represented as CFU/gm of tissue (log_10_) are from triplicate plates. *p < 0.05, **p < 0.005

Immunization with *Mi* also resulted in significantly (*p* < 0.005) increased percentage of KLRG1^hi^ expressing CD8 T cells, as compared to BCG (Fig. 7). KLRG1 is an established marker associated with activated killer CD8 T cells [30]. CD8^+^ T cells generated against HIV and Epstein-Barr virus have been shown to have KLRG1^hi^ expression, thus demonstrating a potential role of *Mi* in elicitation of CD8 T cells with killer ability [31]. Such cells may be beneficial in sensing and liberating the *Mtb* hidden in the macrophages and providing opportunity to activated macrophages to phagocytose and kill it. Furthermore, we observed decline in the *Mtb* burden in the lungs and spleen of the animals that were vaccinated with *Mi*, when compared with placebo (PBS) group. Insignificant change was observed with BCG (Fig. 8). Overall, the results reveal that immunization with *Mi* elicits memory T cell response better than BCG.

## Discussion

TB is declared as a global emergency in the wake of emergence of multidrug resistance of *Mtb*, AIDS-pandemic and non-compliance of BCG vaccine [32–34]. Annually, *Mtb* infects 9 million individuals and kills approximately 2 million people. To control TB epidemic, we require urgent and effective prophylactic and/or therapeutic vaccination strategies. Several clinical trials over the years have shown the inadequacy and inconsistency of BCG vaccine worldwide [35]. Additionally, owing to poor efficacy of BCG in TB endemic areas, there is an urgent requisite to develop a new vaccine against TB [5, 6]. This exigency can be evidenced by the fact that currently there are 14 vaccines in clinical trials. Four of them viz Ad5Ag85A, MVA85A, ChAdOx1.85AMVA85A and MVA85A-IMX313 are in phase I trials; six: RUTI, H56/H4+ IC31, TB/FLU-04 L, ID93 + GLA-SE and MTBVAC are in phase II trials; three: VPM1002, DAR-901 and M72 + AS01E are in phase IIb trials and one: *M. vaccae* in a phase III clinical trial [36, 37].

Several efforts are underway for the development of a novel vaccine candidate to replace BCG or boost its efficacy. Live attenuated or killed whole-cell vaccines (WCVs) against *Mtb* exhibit several advantages over protein adjuvant formulations and recombinant vaccines. WCVs possess broad antigen composition, which includes the complete protein repertoire, lipids, carbohydrates, and other moieties that may elicit immune response and protect against *Mtb*. Live WCVs induce long-lasting immunological memory and have shown great success against diseases like measles, small pox, yellow fever, polio, etc. Therefore, live attenuated vaccines against TB may offer promising results.

In a pursuit for a vaccine that can impart protection against *Mtb*, we did whole genome sequencing, annotation and analysis o*f Mi* to evaluate its safety and suitability for considering it as a vaccine candidate against *Mtb*. The virulence status of the *Mi* was checked and compared with *Mtb* and *Mb* by analyzing differences in the genes belonging to VDD in RAST server. Interestingly, *Mi* contains lesser number of virulent genes as compared to *Mtb* and *Mb*. In addition, it has antigenic proteins similar to *Mtb*. Further, 16 vaccine candidate peptides have been predicted from CD11_6 (*Mi*)*,* making it a suitable vaccine candidate against *Mtb*. Live *Mi* is known to induce abscess, hence we have used heat killed *Mi*. In addition, HK *Mi* induced significantly better generation of both memory CD4 T cells and CD8 T cells [Figs. 6 and 7], which is a fundamental attribute of a successful vaccine. In contrast, main failure of BCG is its inefficiency to induce enduring memory T cells and therefore its potency to protect against TB wanes with age in the vaccinated population [6]. Intriguingly, immunization with *Mi* in the experimental model of TB significantly augmented the generation of central and effector memory response in CD4 and CD8 T cells, as evinced by CD62L^hi^CD44^hi^ and CD62L^lo^CD44^hi^, phenotype respectively. The memory CD4 T cells and CD8 T cells are known to play a fundamental role in maintaining the enduring immunity and protection against *Mtb*. Elicitation of memory CD4 T cells and CD8 T cells by *Mi* may overcome this snag associated with BCG. As compared to placebo group, we observed decline in the *Mtb* load in the lungs and spleen of the mice vaccinated with *Mi.* Based on the memory T cell data, we expect better reduction in *Mtb* growth b*y Mi.* Contrary to our belief, no significant difference was observed in the *Mtb* burden between *Mi* and BCG immunized groups. Currently, it is difficult to explain this discrepancy. However, our laboratory is extensively working to bolster the efficacy of *Mi* against *Mtb* by changing the dose and the route of inoculation. Overall, the *in-silico* and in-vivo studies suggest *Mi* as a potential future vaccine candidate against *Mtb.*

## Conclusion

This study highlights the capability of CD11_6 strain of *Mi* to induce the generation of protective memory T cell response against *Mtb*. Using genome mining and annotation results, we depicted the presence of similar genes in *Mi* encoding for surface membrane antigens, membrane transport and cytosolic proteins as of *Mtb*. The antigenic repertoire of *Mi* indicated that it has enough potential to protect against *Mtb*. We also supported the results with in-vivo experiments; wherein we reported significant increase in the elicitation of memory CD4 T cells and CD8 T cells upon immunization of animals with *Mi*. In essence, the present study signifies that *Mi* has the ability to generate effective memory T cell response against *Mtb* along with eradication of *Mtb* from infected organs. Our results demonstrate that *Mi* has a potential to be used as a vaccine candidate against *Mtb* but extensive future studies are required to gain a deeper understanding in the mechanism of generation of memory T cell response.

## Methods

### Isolation of *Mi*, DNA extraction, whole genome sequencing, assembly and annotation

Strain CD11_6 was isolated from the duodenal mucosa of a CD patient. The patient was tissue transglutaminase (tTG) IgA-antibody (Ab) positive (> 100 U/ml) with abdominal pain and pain while stool passing. For a purpose to check microbial diversity in duodenum, biopsy samples were collected during endoscopy from the patient at Postgraduate Institute of Medical Education and Research, Chandigarh, India. The biopsy sample was homogenized in sterile phosphate saline (PBS) and centrifuged at 4000 rpm for 2 min to remove debris. The supernatant was serially diluted to plate on to tryptic soy agar (TSA; HiMedia, Mumbai, India) and the plates were incubated aerobically at 37 °C for 36 h. All individual colonies appearing on the TSA plates were picked and further passaged on to fresh TSA plates to obtain pure colonies. Thereafter, DNA was isolated from the cell mass of pure colonies and 16S rRNA gene sequencing was used to identify microbes. From the patient coded as CD11, total 7 microorganisms were identified, *Staphylococcus haemolyticus* CD11_1, *Pseudomonas monteilii* CD11_2, *Microbacterium oleivorans* CD11_3, *Janibacter melonis* CD11_4, *Dietzia cinnemea* CD11_5, *Mycobacterium immunogenum* CD11_6 and *Methylobacterium populi* CD11_7. All the strains were sequenced for the purpose of knowing their probable role in pathogenesis of CD. Genomic DNA isolation and amplification was performed as described earlier [38]. Pairwise 16S rRNA gene sequence identity levels were measured by using EzTaxon online server [39]. Mega version 6.0 software was used to create a phylogenetic tree as per the methods described previously [13, 40]. The reference genomes of *Mtb* strain H37Rv and *M. bovis* (*Mb*) strain AF2122/97 with accession numbers NC_000962.3 and NC_002945.4 respectively, were downloaded from NCBI.

Illumina HiSeq 2 × 100 platform was used at C-CAMP to sequence the draft genome of strain CD11_6 (http://ccamp.res.in) next-generation genomics facility, Bengaluru, India. Library preparation and sequencing were performed according to methods described previously [13]. CLC Genomics Workbench (v7.5.1, CLCbio, Arhus, Denmark) was used for de novo assembly.

Rapid Annotation Subsystem Technology (RAST) server was used to annotate genomes [41–43]. RNAmmer 1.2 was used for identifying ribosomal RNA genes in the genomes [44]. The tRNA and tmRNA genes were identified by ARAGORN [45]. For identifying genome sequence similarity in three genomes, Blast Ring Image Generator (BRIG) software was employed [46]. Comparative genomics approach is based on RAST, an automatic annotation server to predict genes involved for a given function (subsystem). Initially, *Mi* was compared with *Mtb* and then with *Mb* for presence of common and unique genes. The comparative data from three genomes were manually compared, as described previously [13]. More than one person independently validated the manual comparison.

### Sequence homology search between the vaccine candidate peptides of *Mtb* and proteome of *Mi*

As described by Vizcaino *et. al*., surface and secretory proteins of virulent strains of H37Rv-*Mtb* are sufficiently immunogenic and therefore can be exploited as a potential vaccine candidate [20]. Similar peptides were present in *Mi* strain CD11_6. Surface or secreted proteins are good targets of the immune system. They are considered useful and important in vaccine design. Vizcaino et al. had described 26 vaccine peptides candidates from virulent strains of H37Rv-*Mtb.* For our in silico study, we had considered these 26 peptides as query sequences to search homologous epitopes in the proteome of *Mi*. Thus, BLAST search tool of RAST was used to explore presence or absence of such peptides in the annotated protein sequences of strain *Mi*. Therefore; sequence homology was determined between the vaccine peptides and proteins of strain CD11_6. The homologous peptides in predicted proteome of *Mi* are inferred as the peptide candidates responsible for providing protection against *Mtb*.

### Antibodies and reagents

All standard chemicals and reagents used in the study were purchased from Sigma (St. Louis, Mo., USA), and fluorochrome labeled Abs to CD4-FITC, CD8-APCCy7, CD44-PerCPCy5.5, CD62L-APC and KLRG1-PE were obtained from BD Biosciences (San Diego, CA), unless indicated. RPMI-1640 and FCS were purchased from GIBCO (Grand Island, NY). For culturing of cells, tissue culture grade plastic-ware was purchased from BD Biosciences (Bedford, MA). Purified protein derivative (PPD) of *Mtb* was prepared by standard protocols, as described elsewhere [47, 48].

### Culturing of *Mtb* H37Rv and *Mi* strain CD11_6

Virulent *Mtb* strain (H37Rv) was was a kind gift from Dr. V. M. Katoch, National JALMA Institute for Leprosy and Other Mycobacterial Diseases, Agra, India. *Mtb* was cultured in Middle brook 7H9 broth (Difco, Sparks, MD) containing glycerol (0.2%) and Tween-80 (0.05%) supplemented with albumin, dextrose and catalase. Viability of bacteria was checked by counting the number of colony-forming units (CFU) by plating onto Middle brook 7H11 medium (Difco, Sparks, MD) supplemented with oleic acid, albumin, dextrose and catalase. *Mi* was cultured in nutrient broth (Himedia, Mumbai, India) at 30 °C for 48 h and number of viable bacteria were determined by plating on nutrient agar plates (Himedia, Mumbai, India).

### Immunization and infection

Female C57BL/6 mice (6–8 weeks) were procured from the animal facility of the CSIR-Institute of Microbial Technology, Chandigarh, India. Animals (4–5 mice/group) were immunized with heat killed *M. immunogenum* (HK*Mi*) (1 × 10^6^ bacilli/ animal). The bacteria were diluted in phospate buffered saline (PBS) and administered in animals via subcutaneous route (s.c.) on base of tail. The control groups were also included comprising of mice inoculated with BCG (positive control) and *placebo* PBS (negative control). After 15 days, a booster dose of *Mi* was administered. Later, animals were aerosol challenged with *Mtb* (100 CFU/mouse) after 30 days using Inhalation Exposure System (Glas-Col, LLC, Terre Haute, IN). After 45 days of infection, animals were sacrificed by cervical dislocation. The lymphocytes were isolated from lungs for immunological assays. Further, *Mtb* load was enumerated in the lungs and spleen by CFU counting.

### Cell culture and expression of memory markers on T cells by flowcytometry

Lungs obtained from experimental and control mice were perfused by chilled PBS, fragmented into small pieces and digested with collagenase (2 mg/ml) for 30 min/37 °C. The RBCs were lysed with ACK lysis buffer (150 mM NH_4_Cl, 1 mM KHCO_3_ and 0.1 mM Na_2_EDTA, pH 7.4). The cell viability was measured by trypan blue exclusion method recorded as > 99%. The lymphocytes isolated from lungs (2 × 10^5^ cells/well) were cultured in 96 well U-bottomed microtiter culture plates with RPMI 1640 + FCS 10% (200 μl) and purified protein derivative (PPD; 25 μg/ml) for 48 h/37 °C/5% CO_2_. Later, cells were harvested and washed and stained with fluorochrome labeled Abs to CD4, CD8, CD44, CD62L, CD127 and KLRG1 for 1 h/4 °C. Standard protocols of washing/incubation were followed at each stage. Samples were acquired on FACS-Aria III and analyzed using FACS DIVA software (BD Biosciences, San Jose, CA).

### Enumerating protection efficacy of *Mi* by CFU counting

The lung and spleen homogenates were prepared and plating was done on 7H11 agar plates with 10-fold serial dilutions. The plates were incubated at 37 °C/5% CO_2_ and colonies were enumerated after three weeks of incubation. Results are expressed as log_10_ colony-forming units (CFUs) per gm of lung tissue.

### Statistical analysis

Graph Pad Prism software program was used to perform all statistical analysis. Statistical testing was performed by one way ANOVA for group analysis and Student’s t-test was used for comparing two groups.

## Abbreviations

BRIG: BLAST Ring Image Generator
CD: Celiac disease
CFU: Colony-forming units
HK*Mi*: Heat killed *M. immunogenum*
*Mb*: *Mycobacterium bovis*
*Mi*: *Mycobacterium immunogenum*
*Mtb*: *Mycobacterium tuberculosis*
PBS: Phosphate buffered saline
PPD: Purified protein derivative
RAST: Rapid Annotation using Subsystem Technology
TB: Tuberculosis
VDD: Virulence, disease and defense
